# Elmidae (Coleoptera, Byrrhoidea) larvae in the state of São Paulo, Brazil: Identification key, new records and distribution

**DOI:** 10.3897/zookeys.151.1879

**Published:** 2011-12-03

**Authors:** Melissa Ottoboni Segura, Francisco Valente-Neto, Alaíde Aparecida Fonseca-Gessner

**Affiliations:** 1 Programa de Pós-Graduação em Ecologia e Recursos Naturais, Universidade Federal de São Carlos, São Carlos, São Paulo, Brazil; 2 Departamento de Hidrobiologia, Universidade Federal de São Carlos, São Carlos, São Paulo, Brazil

**Keywords:** riffle beetles, streams, aquatic insects, illustrated key

## Abstract

The family Elmidae Curtis, 1830 has cosmopolitan distribution and most species inhabit riffles on streams and rivers, hence the name “riffle beetle”. In recent years, this family has been featured in papers addressing the assessment and environmental monitoring of water quality. In Brazil, studies on the family remain scarce and the present investigation is a pioneering study in the state of São Paulo. This study aims to propose a taxonomic key for the identification of larvae of Elmidae genera known to occur in the State, as well as to report new records and the distribution of these genera. The material analyzed was collected from various locations in each of 15 drainage basins from 2005 to 2010. The identification key includes 12 genera (*Austrolimnius* Carter & Zeck, 1929, *Heterelmis* Sharp, 1882, *Hexacylloepus* Hinton, 1940, *Hexanchorus* Sharp, 1882, *Huleechius* Brown, 1981, *Macrelmis* Motschulsky, 1859, *Microcylloepus* Hinton, 1935, *Neoelmis* Musgrave, 1935, *Phanocerus* Sharp, 1882, *Potamophilops* Grouvelle, 1896, *Stegoelmis* Hinton, 1939 and *Xenelmis* Hinton, 1936) known in Brazil as well as three morphotypes designated herein as Genus A, Genus M and Genus X. The genus *Hexanchorus* is recorded for the first time in the state of São Paulo.

## Introduction

Elmidae Curtis, 1830, is a truly aquatic beetle family with cosmopolitan distribution. Most species are found mainly in areas of riffles in lotic ecosystems (rivers and streams). The genera of this family are distributed in two subfamilies: Elminae Curtis, 1830 and Larainae LeConte, 1861 (Jäch and Balke 2008). Among aquatic beetles, Elmidae is the fourth most speciose family, with around 1330 species distributed in 146 genera throughout the world (Jäch and Balke 2008). In the Neotropical region, there are 330 known species in 44 genera, of which 250 species and 39 genera are recorded in South America (Manzo 2005; Passos et al. 2007; Maier and Spangler 2011). In Brazil, there are checklists for the Amazonian region and state of Rio de Janeiro, with 59 recorded species (Passos et al. 2009; Passos et al. 2010).

Elmid larvae and adults generally exploit the same habitats and obtain food by scraping the surface of rocks, wood fragments, roots and leaves, consuming periphyton and detritus (Seagle 1982) and have been included among the herbivores (Leech and Chandler 1956; Brown 1972). However, Seagle (1982) reclassified this family as detritivorous-herbivorous. In terms of functional feeding groups, elmids have been described as scrapers, collectors/gatherers and/or shredders (White and Brigham 1996; Cummins 1973).

Larval development involves five to eight instars and the life cycle can last from six months (Brown 1987) to six years (Steedman and Anderson 1985), depending on temperature and quantity and quality of food available (Brown 1987). At the end of the last instar, the larvae generally migrate to the banks of lotic systems and pupate. In some cases, the larvae remain on their original substrate and pupate in situ when the water level falls (White and Jennings 1973; White 1978; Seagle 1980).

The family is used in monitoring programs and environmental assessments because of the sensitivity of most species to physical and chemical changes in the environment (Ribera and Foster 1992; Ribera 2000; Garcia-Criado and Fernandez-Aláez 2001; Compin and Céréghino 2003). However, in the Neotropical region the use of this family in environmental assessments is hindered by the lack of information on immature forms, and the availability of identification keys and reference collections.

Although a number of authors have invested effort in the collection of taxonomic data on the group, particularly the description of species (Hinton 1936, 1937, 1939, 1940, 1945, 1971, 1972, 1973; Brown 1970,^ 1971^, ^1981^; Spangler and Santiago 1987; Spangler 1966, 1990; Costa et al. 1988) literature about the Brazilian fauna remains scarce. In recent years, some South American researchers have intensified studies on Elmidae biology (Costa et al. 1988; Passos et al. 2003a), ecology (Costa et al. 1988; Passos et al. 2003b; Segura et al. 2007a, b) and taxonomy, including the description of new species (Passos and Felix 2004a, b; Manzo 2006; Archangelsky and Manzo 2006, 2007; Archangelsky et al. 2009; Vanin and Costa 2011) and identification keys (Passos et al. 2007; Manzo 2005; Manzo and Archangelsky 2008; Mugnai et al. 2010). However, the studies carried out by Manzo (2005) and Manzo and Archangelsky (2008) include few specimens of Elmidae from Brazil. In contrast, Passos et al. (2007) and Mugnai et al. (2010) offer taxonomic keys for the family Elmidae in Brazil, more specifically, in the state of Rio de Janeiro. Table 1 lists the genera of Elmidae recorded in South America and known genera for Brazil and the southeastern region of Brazil.

The aim of this paper is to propose an identification key for the genera of elmid larvae in the state of São Paulo, Brazil, based on the study of material from various aquatic ecosystems located in various vegetation types.

## Materials and methods

The majority of specimens examined were collected from 2005 to 2010, by different sampling methods in expeditions of the BIOTA-FAPESP project: Survey and Biology of Aquatic Insecta and Oligochaeta of Lotic Systems in the State of São Paulo (Process number 2003/10517-9). The material is deposited in the collection of the Aquatic Insect Laboratory of the Universidade Federal de São Carlos and the São Paulo Museum of Zoology of the Universidade de São Paulo (Brazil).

**Figure 1.**
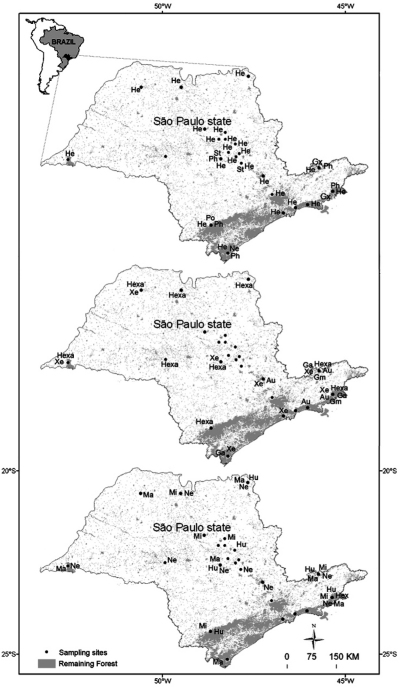
Distribution of Elmidae in the state of São Paulo. Codes: Au= *Austrolimnius* Ga= Genus A Gm= Genus M Gx= Genus X He= *Heterelmis* Hexa= *Hexacylloepus* Hex= *Hexanchorus* Hu= *Huleechius* Ma= *Macrelmis* Mi= *Microcylloepus* Ne= *Neoelmis* Ph= *Phanocerus* Po= *Potamophilops* St= *Stegoelmis* Xe= *Xenelmis*.

The material was collected from 52 aquatic systems in each of 15 drainage basins in the state of São Paulo (Fig. 1) in areas of different vegetation types, cerrado (Brazilian savannah), Atlantic rainforest and seasonal semi-deciduous forest, and in areas dominated by extensive monoculture (mainly sugarcane, banana and eucalyptus plantations) and pasture : Araraquara city region (21°50'S, 48°08'W); Campos do Jordão, Parque Estadual de Campos do Jordão (22°41'S, 45°29'W); Capão Bonito, Parque Estadual de Intervales (24°16'S, 48°27'W); Gália, Estação Ecológica de Caetetus (22°23'S, 49°41'W); Luis Antônio, Estação Ecológica Jataí (21°36'S, 47°48'W); Pedregulho, Parque Estadual das Furnas do Bom Jesus (20°13'S, 47°27'W); Santa Rita do Passa Quatro, Parque Estadual de Vassununga (21°38'S, 47°37'W); São Carlos city region (22°02'S, 47°46'W); São José do Rio Preto (20^o^33'S, 49^o^14'W); São Luiz do Paraitinga, Parque Estadual da Serra do Mar – Núcleo Santa Virgínia (24^o^20'S, 45^o^07'W); São Paulo city region (23°19'S, 46°51'W); Teodoro Sampaio, Parque Estadual do Morro do Diabo (22°36'S, 52°18'W); Ubatuba, Parque Estadual da Serra do Mar – Núcleo Picinguaba (23^o^22'S, 44^o^46'W) Jundiaí (23°45'S, 46°56'W) Cananéia (24°50'S, 48°14'W).

**Figure 2.**
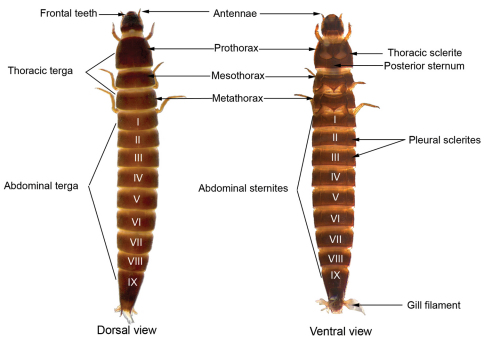
General morphology scheme of larvae of the genus *Macrelmis* showing most of the characters used to identify elmid larvae.

Only mature larvae (larger, well-sclerotized larvae with functional spiracles) were used to build the genus identification key. The traits used to identify the larvae were based on Hinton (1940), Spangler and Santiago-Fragoso (1987, 1992), Passos et al. (2007) and Manzo and Archangelsky (2008) (Fig. 2).

The images used in the identification key were taken with a Leica DFC 280 camera coupled to a Leica MZ9_5_ stereomicroscope. The images were treated with Adobe Photoshop CS4 to correct contrast, brightness and imperfections.

**Table 1.** List of genera reported in the literature for South America, Brazil and Southeastern Brazil. (*) Genera whose larvae are Unknown and (■) First record for the State of São Paulo.

**Genera**	**South America**	**Brazil**	**Southeastern**
**Subfamily Elminae**			
*Austrelmis* Brown, 1984	X		
*Austrolimnius* Carter & Zeck, 1929	X	X	X
*Cylloepus* Erichson, 1847	X	X	X
Epodelmis* Hinton, 1973	X		
Gyrelmis* Hinton, 1940	X	X	
*Heterelmis* Sharp, 1882	X	X	X
*Hexacylloepus* Hinton, 1940	X	X	X
Hintonelmis* Hinton, 1971	X	X	
Holcelmis* Hinton, 1973	X		
*Huleechius* Brown, 1981	X	X	X
Jolyelmis*Spangler & Faitoute, 1991	X		
*Luchoelmis* Spangler & Staines, 2001	X		
*Macrelmis* Motschulsky, 1859	X	X	X
*Microcylloepus* Hinton, 1935	X	X	X
*Neoelmis* Musgrave, 1935	X	X	X
Neolimnius*Hinton, 1939	X	X	
Notelmis* Hinton, 1941	X		
Onychelmis*Hinton, 1941	X		
Oolimnius* Hinton, 1939	X	X	
Pagelmis*Spangler, 1981	X		
*Phanoceroides* Hinton, 1939	X	X	
*Pilielmis* Hinton, 1971	X	X	X
Portelmis* Sanderson, 1953	X	X	
*Stegoelmis* Hinton, 1939	X	X	X
Stenhelmoides* Grouvelle, 1908	X	X	X
*Stethelmis* Hinton, 1945	X		
Tolmerelmis* Hinton, 1972	X	X	
Tyletelmis* Hinton, 1942	X	X	
*Xenelmis* Hinton, 1936	X	X	X
**Subfamily Larainae**			
*Disersus* Sharp, 1882	X		
*Hexanchorus*^■^ Sharp, 1882	X	X	X
*Hydora* Brown, 1982	X		
*Hypsilara* Maier & Spangler, 2011	X		
Neblinagena* Spangler,1995	X		
*Phanocerus* Sharp, 1882	X	X	X
*Pharceonus* Spangler & Santiago, 1992	X		
*Potamophilops* Grouvelle, 1896	X	X	X
*Pseudodisersus* Brown, 1981	X		
*Roraima* Kodada & Jach, 1999	X		
**Total**	**39**	**23**	**15**

## Results and discussion

This paper proposes an identification key for larvae of Elmidae at the genus level, encompassing 12 genera previously recorded in South America: *Austrolimnius* Carter & Zeck, 1929, *Heterelmis* Sharp, 1882, *Hexacylloepus* Hinton, 1940, *Hexanchorus* Sharp, 1882, *Huleechius* Brown, 1981, *Macrelmis* Motschulsky, 1859, *Microcylloepus* Hinton, 1935, *Neoelmis* Musgrave, 1935, *Phanocerus* Sharp, 1882, *Potamophilops* Grouvelle, 1896, *Stegoelmis* Hinton, 1939 and *Xenelmis* Hinton, 1936 (Table 1), among which *Huleechius* and *Potamophilops* are included for the first time in an identification key for Brazil and *Hexanchorus* is recorded for the first time in the state of São Paulo. In addition, three morphotypes are identified, denominated herein as Genus A, Genus M and Genus X.

Genus A is morphologically similar to *Heterelmis* based on the description offered by Passos et al. (2007), but is separated from this genus by the arrangement and number of rows of tubercles. Moreover, the mesopleura and metapleura are divided into three partsin *Heterelmis*, but only two parts in Genus A. Genus M is similar to but separated fromGenus X, which has pleural sclerites on abdominal segments I to VII, whereas Genus M has pleural sclerites only on abdominal segments I to IV.

It should be noted that young *Hexanchorus* larvae (Fig. 22) do not yet have the large tubercle found in mature larvae on each side of the midline on abdominal tergum VIII (Figs 19 and 20).

In general, most of the genera were distributed for all regions of State of São Paulo, such as *Heterelmis*, *Hexacylloepus*, *Macrelmis* e *Xenelmis* (Fig. 1). It is worthwhile mentioning that *Heterelmis* was found in both preserved and impacted areas. On the other hand, the distribution of some genera appeared to be restricted to some regions. For instance, *Genus A*, *Genus M*, and *Genus X* were found in streams located at eastern region state of São Paulo, *Hexanchorus* was recorded only in the Coast region and *Stegoelmis* in Central region of the state.

Identification key to larvae of Elmidae (Coleoptera: Byrrhoidea) found in the state of São Paulo, Brazil.

**Table:** 

1	Body strongly flattened dorsoventrally (Fig. 3). Lateral margins of thoracic and abdominal segments with falcate and narrow lateral extensions (Fig. 4). Pleural sclerites present on abdominal segments I-VIII. Length: 4.6–5.5mm	*Phanocerus*
–	Body cylindrical, subcylindrical or slightly flattened dorsoventrally. Lateral margins of thorax and abdominal segments, in general, without lateral extensions; if present, never falcate (Fig. 5). Pleural sclerites present on abdominal segments I–IV or I–VII	2
2	Sensory appendage of second antennomere very long (longer than third antennomere) (Fig. 7). Pairs of median and lateral tubercles arranged in longitudinal rows along the thoracic (except the prothoracic) and abdominal terga. Length: 2.5–3.0 mm (Figs 5 and 6)	*Austrolimnius*
–	Sensory appendage of second antennomere short (shorter than third antennomere) (Fig. 8). Tubercles in the thoracic and abdominal terga not arranged as above	3
3	Abdominal terga with posterior middorsal expansion, in at least four segments (Figs 9 and 11). Pleural sclerites present on abdominal segments I-IV. Length: 2.7–3.5mm (Fig. 10)	Genus M
–	Abdominal terga without posterior expansion as above. Pleural sclerites present on abdominal segments I-VI or I-VII (Fig. 12)	4
4	Prothorax without posterior sternum (procoxal cavity open) (Fig. 13)	5
–	Prothorax with a posterior sternum (procoxal cavity closed) (Fig. 14)	8
5	Pleural sclerites present on abdominal segments I-VII. Larvae densely pubescent. 10 mm (see Vanin and Costa 2011)	*Potamophilops*
–	Pleural sclerites present on abdominal segments I-VI (Fig. 12)	6
6	Ventral region of prothorax with four sclerites: one anterior pair and one posterior pair (Fig. 16). Posterior extremity of last abdominal segment bifurcated (Fig. 17). Body usually curved in “C” (lateral view). Length: 2.6–3.2 mm (Fig. 15)	*Xenelmis*
–	Ventral region of prothorax with seven sclerites: one anterolateral pair, two lateral pairs, and one central sclerite (Fig. 18). Last abdominal segment different from above. Body shape variable	7
7	Posterior margin of abdominal segment VIII with two large laterodorsal tubercles on the tergum (Figs 19 and 20). Lateral margins of all abdominal segments moderately expanded laterally, without spinous processes and with simple setae (Fig. 21). Head usually with six stemmata on each side. Length: 4.2 – 5.3mm	*Hexanchorus*
–	Posterior margin of abdominal segment VIII without laterodorsal tubercles (Fig. 23). Lateral margins of all abdominal segments expanded laterally, with spinous processes bearing numerous ornate setae (Fig. 24). Head usually with one stemma on each side. Length: 6.0 – 7.0mm.	*Stegoelmis*
8	Abdominal terga with middorsal and laterodorsal prominent humps in at least seven segments. Length: 3.2–3.8mm (Figs 25, 26 and 27)	Genus X
–	Abdominal terga without prominent humps (Fig. 28)	9
9	Last abdominal segment long and slender, three times longer than wide (Fig. 28)	10
–	Last abdominal segment variable in shape and length, but not three times longer than wide (Figs 33 and 34)	11
10	Tubercles arranged in a pair of middorsal rows on the thoracic and abdominal terga. Length: 2.7–3.5mm (Figs 28 and 29)	*Hexacylloepus*
–	Tubercle rows absent on middorsal line of the thoracic and abdominal terga. Length: 2.0 – 2.5mm (Figs 30 and 31)	*Neoelmis*
11	Anterior margin of head with a large tooth on each side, between bases of antennae and clypeus (Fig. 32)	12
–	Anterior margin of head without teeth (Fig. 36)	13
12	Body slightly flattened ventrally; thoracic and abdominal segments wider than long. Length: 7.9 – 8.8mm (Fig. 33)	*Macrelmis*
–	Body subcylindrical, not flattened; thoracic and abdominal segments almost as wide as long. Length: 6.0 – 7.2mm (Fig. 34)	*Huleechius*
13	Tubercles randomly distributed on thoracic terga and abdominal segment IX. Tubercles on remaining terga arranged partially in longitudinal rows. Length: 2.4 – 2.7mm (Figs 35 and 36)	*Microcylloepus*
–	Tubercles arranged in eight (Fig. 38) or ten (Fig. 40) longitudinal rows on thoracic and abdominal terga I-VIII	14
14	Tubercles arranged in eight longitudinal rows on the thoracic and abdominal terga (mesothorax and metathorax). Prothorax without rows of tubercles. Mature larvae very sclerotized. Length: 3.5 – 4.5mm (Figs 37 and 38)	Genus A
–	Tubercles arranged in ten longitudinal rows on the thoracic and abdominal terga (mesothorax and metathorax). Eight longitudinal rows of tubercles on prothorax. Length: 4.4 – 5.2mm (Figs 39 and 40)	*Heterelmis*


**Figures 3–4.**
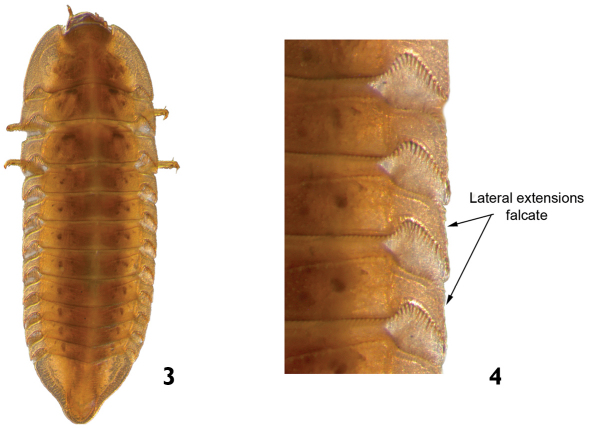
*Phanocerus* sp.: **3** dorsal view **4** dorsal view (detail of the lateral margins of body segments).

**Figures 5–7.**
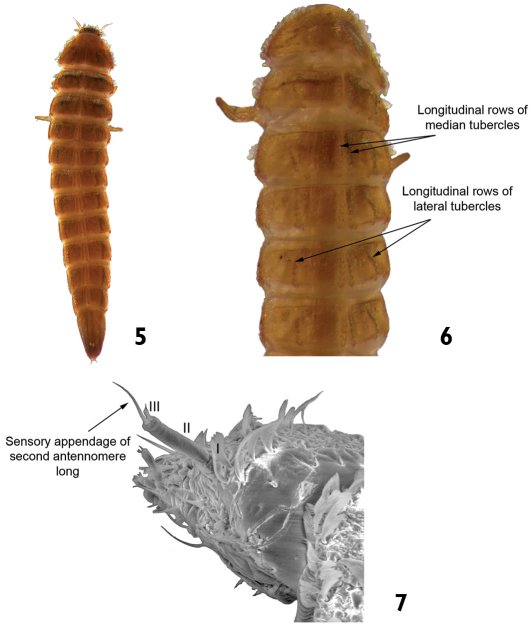
*Austrolimnius* sp.: **5** dorsal view **6** dorsal view (detail of the median and lateral longitudinal rows of tubercles) **7** head (detail of the sensory appendage on the antenna).

**Figure 8.**
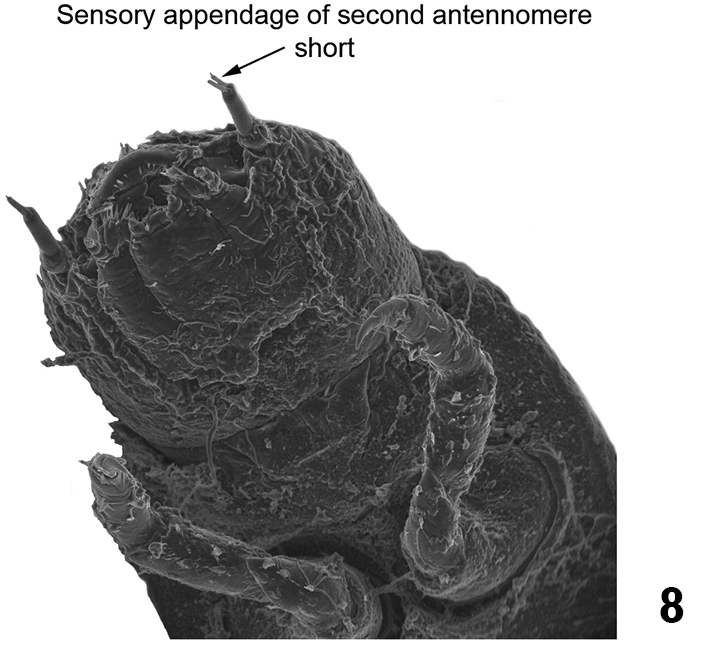
*Heterelmis* sp. ventral view of head and prothorax (detail of the sensory appendage on the antenna).

**Figures 9–11.**
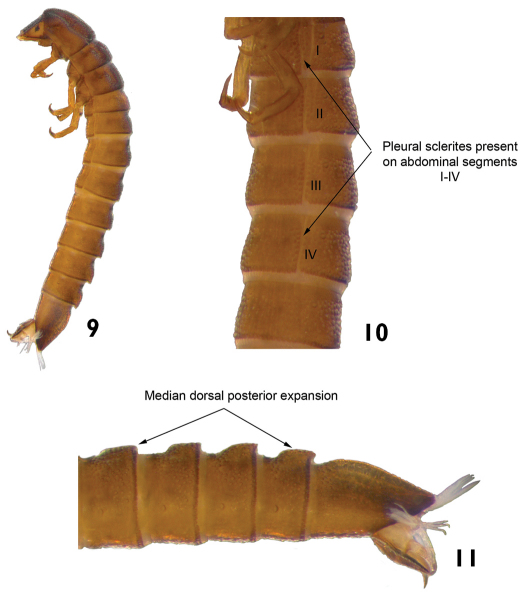
Genus M: **9** lateral view **10** lateral view (detail of the pleural sclerites) **11** lateral view (detail of the last abdominal segments)

**Figure 12.**
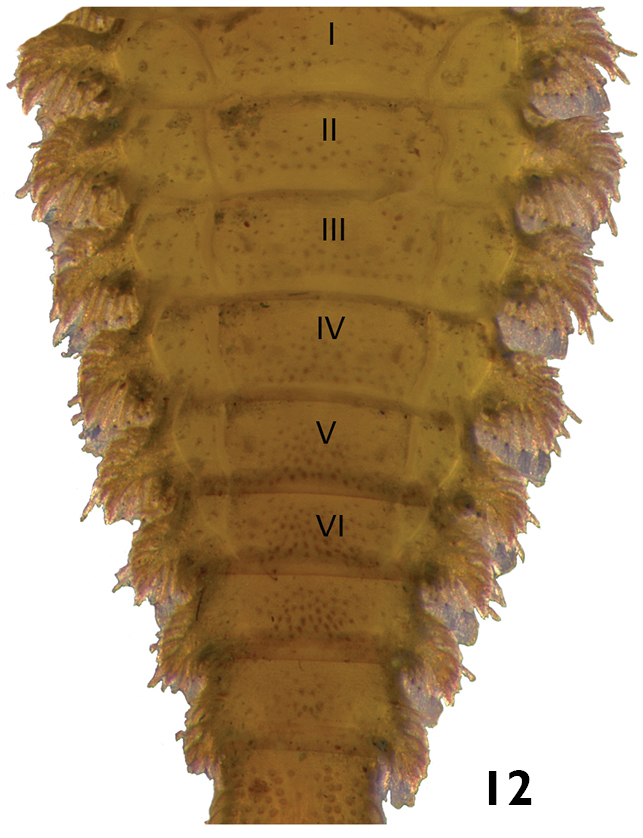
*Stegoelmis* sp. ventral view (detail of the pleural sclerites).

**Figure 13–14.**
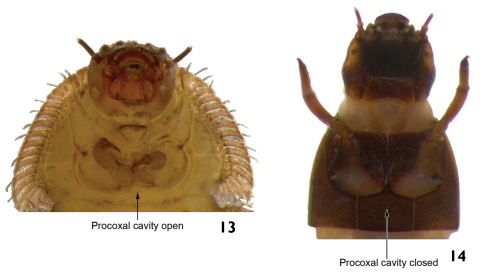
Procoxal cavities. **13** *Stegoelmis* sp. ventral view (detail of the prothorax) **14** *Macrelmis* sp. ventral view (detail of the prothorax).

**Figures 15–17.**
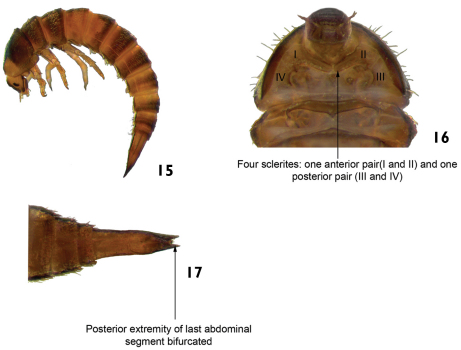
*Xenelmis* sp. **15** lateral view **16** ventral view (detail of the prothorax) **17** ventral view (detail of the last abdominal segment).

**Figure 18.**
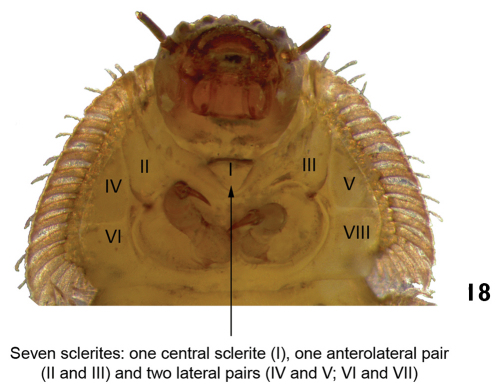
*Stegoelmis* sp. ventral view (detail of the prothorax).

**Figures 19–22.**
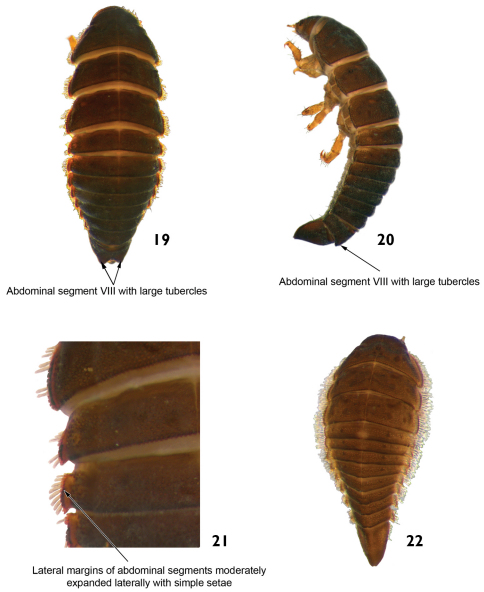
*Hexanchorus* sp.: **19** dorsal view **20** lateral view **21** dorsal view (detail of the lateral margins of abdominal segments) **22** dorsal view (early larva).

**Figures 23–24.**
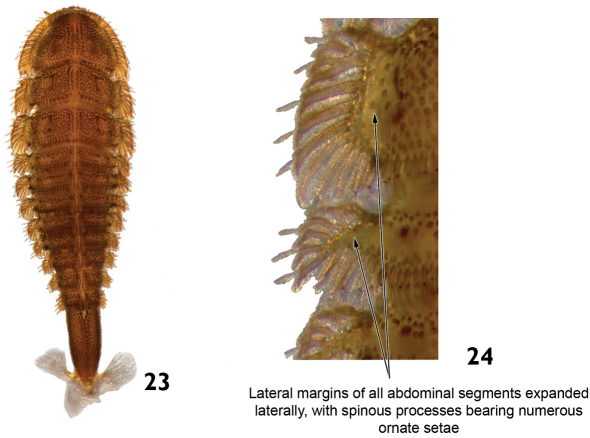
*Stegoelmis* sp.: **23** dorsal view **24** dorsal view (detail of the lateral margins of abdominal segments).

**Figures 25–27.**
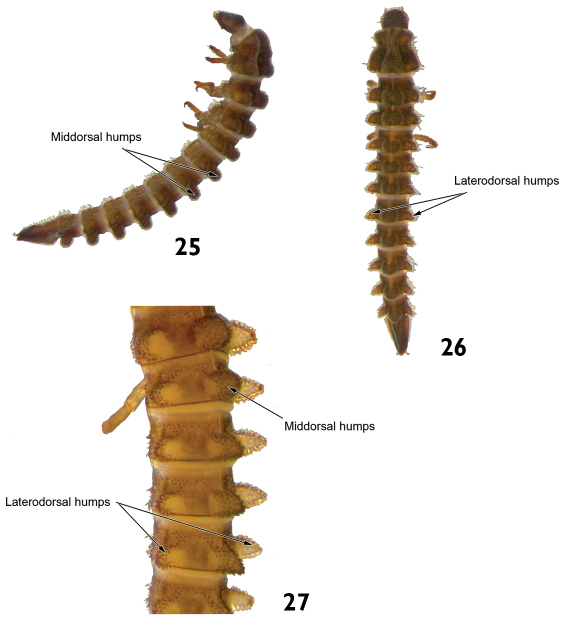
Genus X: **25** lateral view **26** dorsal view **27** lateral view (detail of the abdominal terga).

**Figures 28–29.**
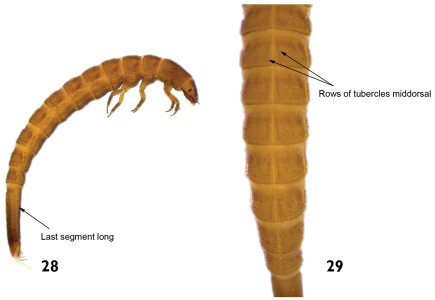
*Hexacylloepus* sp.: **28** lateral view (detail of the last abdominal segment). **29** dorsal view (detail of the midline).

**Figures 30–31.**
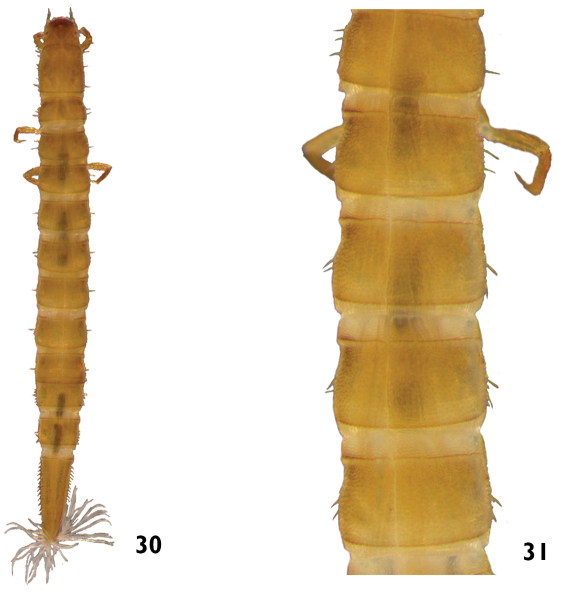
*Neoelmis* sp.: **30** dorsal view **31** dorsal view (detail of the midline).

**Figure 32.**
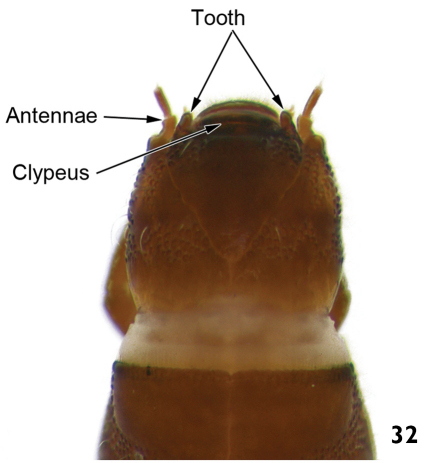
*Macrelmis* sp. dorsal view of the head (detail of the anterior margin).

**Figures 33–34.**
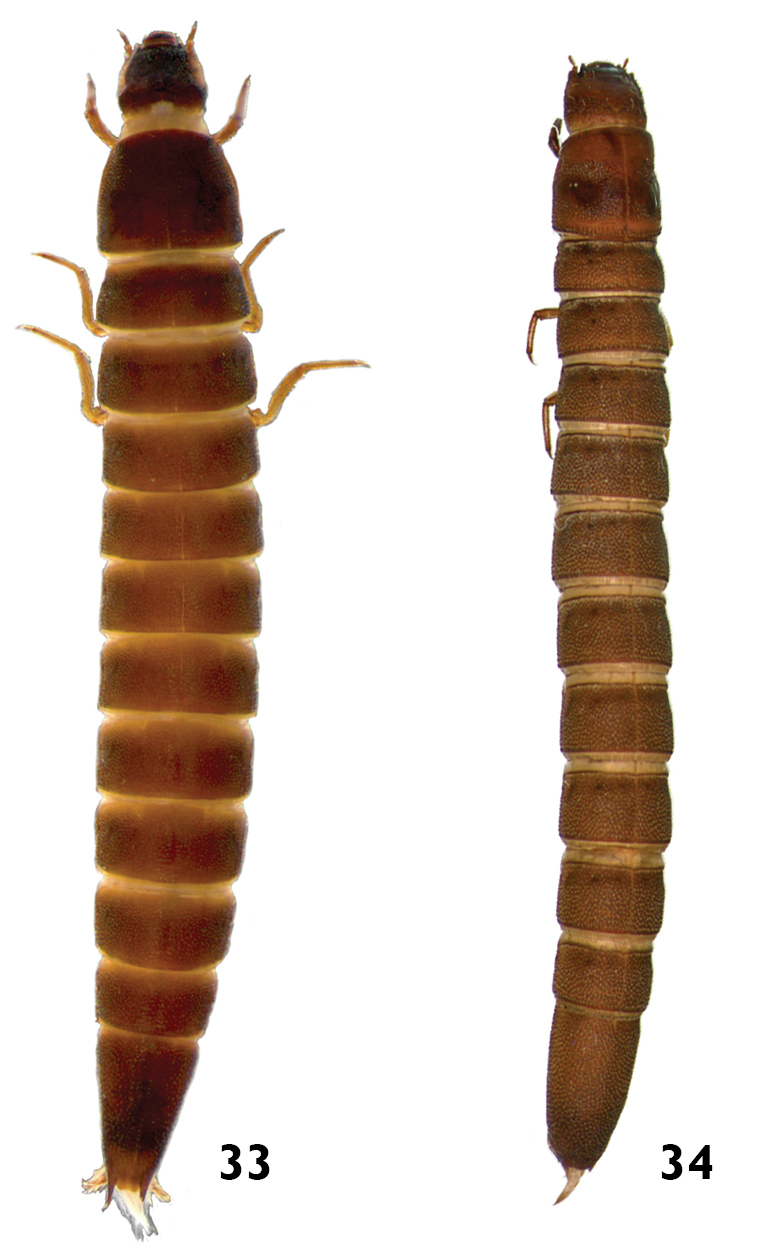
**33** *Macrelmis* sp. dorsal view **34** *Huleechius* sp. dorsal view.

**Figures 35–36.**
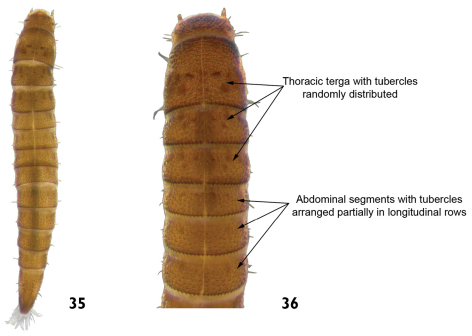
*Microcylloepus* sp.: **35** dorsal view **36** dorsal view (detail of the thoracic and abdominal terga).

**Figures 37–38.**
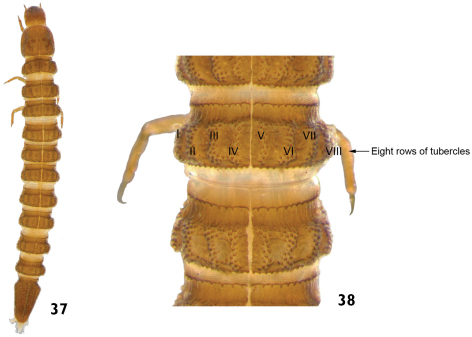
Genus A: **37** dorsal view **38** dorsal view (detail of the thoracic and abdominal terga).

**Figures 39–40.**
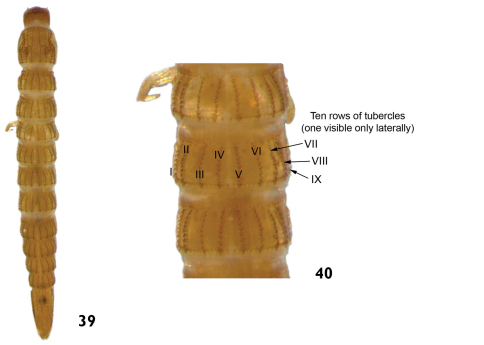
*Heterelmis* sp.: **39** dorsal view **40** dorsal view (detail of the thoracic and abdominal terga).
